# Sexual Transmission of Hepatitis C Virus Between HIV Infected Subjects and Their Main Heterosexual Partners

**DOI:** 10.5812/hepatmon.13593

**Published:** 2013-11-05

**Authors:** Abbas Alipour, Abbas Rezaianzadeh, Jafar Hasanzadeh, Abdorreza Rajaeefard, Mohammad Ali Davarpanah

**Affiliations:** 1 Community Medicine Department, School of Medicine, Mazandaran University of Medical Sciences, Sari, IR Iran; 2 Epidemiology Department, School of Health and Nutrition, Research Center for Health Sciences, Shiraz University of Medical Sciences, Shiraz, IR Iran; 3 HIV Research Center, Shiraz University of Medical Sciences, Shiraz, IR Iran

**Keywords:** HIV, Hepatitis C Virus, Transmission

## Abstract

**Background:**

Overall, 60-70% of the hepatitis c virus (HCV) transmission routes is parenteral, and in 30-40% of the cases is unknown (e.g. sexual route). Knowing these routes in HIV infected dyads is very important due to clinical and methodological reasons.

**Objectives:**

The present study aimed to identify and quantitatively investigate HIV-infected individuals and their main heterosexual partners regarding the risk factors of HCV transmission.

**Patients and Methods:**

One hundred sixty eight of 984 couples were chosen through random generated numbers using a computer program from behavioral consultation center in Shiraz, Iran. We used actor partner independent model (APIM) and multilevel analysis to assess multiple risk factors for HCV, while partitioning the source of risk at the individual and couple levels.

**Results:**

Age of the index samples was 38.71 ± 7 years, and 33.2 ± 6.3 for their main heterosexual partners; the mean duration of sexual relationship for couples was 11.9 (median = 8.5) years. Multivariate analysis showed that actor risk factor of intravenous drug using (IDU) (AOR= 13.03; 95% CI: 3.9- 43.82) and actor cofactors of HIV positivity (AOR = 7.1; 95% CI: 1.37- 36.97), razor sharing (AOR = 4.81; 95% CI: 1.84- 12.55), sex (AOR = 8.83; 95% CI: 3.16- 24.87), and condom use in sexual activity with main partner (AOR = 0.15; 95% CI: 0.02- 0.44) were associated with actor HCV positivity.

**Conclusions:**

Health care providers need to pay special attention to sexual transmission of HCV among HIV-infected individuals, and should recommend control/preventive measures for HCV sexual transmission.

## 1. Background

In spite of the importance and scope of HCV infection, no agreement has been reached regarding the transmission routes of this virus (1). Overall, 60-70% of the virus transmission routes are parenteral ones; however, the transmission routes have not been identified in 30-40% of the cases (1-5). Among the transmission routes, sexual ones have been both vague and important. One of the major problems of the previous studies conducted on the issue is the method used for investigating the risk factors of HCV (6). In general, each individual living in today’s risky society deals with a number of individual factors (level 1) as well as several factors which are related to one’s marital life (couple level, level 2). In fact, only a limited number of studies have separately investigated the individual and couple level intra familial risk factors of the virus (7). Overall, one way to investigate the transmission routes of the virus is conducting studies on heterogeneous couples, since such individuals have had close relationships with each other for a long period of time (7, 8). This kind of investigation is of great importance particularly in special target populations (such as HIV infected couples) with a high prevalence of HCV since such heterogeneous couples are faced with several risk factors, such as shared injection and sexual instruments, both inside and outside their families. In addition, after controlling the effect of other risk factors, this investigation method can clearly show the role of sexual transmission route of HCV, as well (7). HCV transmits through sexual route in these dyads is controversy (9-13), and it seems that the methodology and analysis of past studies had important role in their interpretation (6, 7).

In addition to methodology, investigation of the transmission routes of HCV infection in the Iranian HIV-infected population is highly important from two other perspectives. First, getting infected by HCV is proposed as an important cofactor in immunological as well as clinical improvement, and the treatment results of the HIV-infected individuals (14-17). In addition, according to the investigations, injection with shared instruments has been found to be the transmission route of HIV in 70% of the cases in Iran (18). The prevalence of HCV infection is also quite high among the injection drug users (above 70%). In spite of applying preventive strategies in the recent years, the rate of HIV transmission is still high among injection drug users (19). On the other hand, sexual transmission is increasing among all the groups and it is believed that due to the risky sexual behaviors, Iran is at risk of the third wave of HIV infection (20). Besides, the sexual partners of the Injection drug users, as members of the bridging population, play a major role in transmitting HIV and HCV from this risky group of the society (20, 21).

## 2. Objectives

Therefore, the present study aimed to identify and quantitatively investigate HIV-infected individuals and their main heterosexual partners regarding the risk factors of HCV transmission in both individual and dyadic levels in Shiraz (southern of Iran). The researchers also intended to present appropriate strategies for controlling the transmission of this infection.

## 3. Patients and Methods

HIV infected persons and their main sexual partners were recruited from the Behavioral Consultation Center in Shiraz, Iran in 2011. This center is located in Shiraz (center of Fars province) South of Iran with a population of 1200000. These individuals were diagnosed as definite HIV infection (by serial ELISA and Western blot tests) that were picked up among thousands of individuals referred to this center due to risky behaviors from Fars province. In the center, HIV infected patients receive some frees services such as regular laboratory and radiological examinations, any medications prescribed by physicians, and personal and familial consultation. We took into account the following criteria to choose samples (dyads); age over 17 years, sexual relationship over 1 year, and having at least one sexual contact with each other in the current month. 

Of the 1338 dyads enrolled in the center, 984 had eligible criteria and verbal consent to enter the study. One hundred sixty eight of 984 dyads were chosen through random generated numbers using a computer program. Index case in each dyad was considered as actor, and their main heterosexual partner was considered as partner.

A formal written consent was obtained from each participant. The interviewees were assured that their identities would be remained anonymous. To achieve this, each questionnaire was linked to a blood sample by a unique code number, which was assigned to each study participant. The interviews were conducted by a trained interviewer using standard Behavioral sero survey questionnaire. Questionnaires were completed simultaneously by sex-matched interviewers for male and female partners in separate offices. Before completion of the main part of questionnaire, its preliminary part was completed for verification of their main partnership. The questionnaire measures behavioral risk factors (e.g. drug use, IDU, risky IDU), cofactors or biologic effect modifiers (e.g. age, sex, HIV positivity) which moderate the risk of HCV infection in the context of a given risk behavior and social risk factors (incarceration, low education). Besides, individual risk factors and cofactors, couple level (level 2) risk factors which represent sources or moderators of HCV infection specific to the main partnership (e.g. Condom use with main partner, partnership duration) were assessed too.

Regarding specimen collection, each interviewee consented to have a whole blood test using 5 ml whole blood samples. Each partner was tested for anti-HIV, and antigen of HIV, Total Leukocyte Count (TLC), and Anti-HCV antibody. Each person was tested for anti-HCV and TLC. Anyone who had a repeated positive result with the first ELISA kit (Delaware Biotech, The USA), and sequentially another positive result by a western blot (Diagnostic, Germany) was considered as HIV positive. A participant who had a positive result with the third generation of ELISA kit (Dia.Pro, Italy) was considered as HCV positive. All HIV positive participants were tested for CD4 count. Measurement of CD4 counts was performed through flow cytometry technique (Partec, Germany). All the laboratory personnel and processors were blinded to behavioral information.

### 3.1. Statistical Analysis

A priori sample size of 288 subjects was calculated based on a 25% prevalence of HCV risk factor (nearest proportion to 50% in a pilot study) in their partners within a ± 5% margin of error. In multilevel analysis and with assumption of 2 subjects in each cluster (dyad), intra class correlation of 0.2 (pilot study), 168 dyads were calculated (22).

Because in our study individual nested in dyads (hierarchically structure) and the observation collected from each person were dependent, and the assumption of independency of persons were not met; therefore, the conventional methods of inferential data analyses, such as regression analyses could not be used. In this kind of clustering, each cluster or dyads consisted of an actor (the person who respond to question), and a partner (the person who is in relationships with). Persons in each dyadic relationship influence each other (mutual influence). One of the best models which can measure and test this mutual effect is actor partner interdependence model (APIM) (23).

Besides interdependency of actor and partner, small group size (two people in each cluster) is another problem for analyzing APIM. Multilevel analytic techniques have been developed and applied to resolve these problems. We used proc NLMIXED procedure (SAS software; version 9.1; SAS institute) which delivers maximized and theoretically exact integrated likelihood estimates based on an adaptive Gaussian quadrature (23, 24). Before using conditional multilevel models, we estimated Pearson type intraclass correlation coefficient (PICC) for assessment of interdependency. According to Meyers recommendation, a liberal test (P < 0.2, two tailed) should be used for interdependency test (25). We used Holm-Sidak correction for adjusting P values in state in which we conduct large number of significant tests. Not only this method guards against family wise error, but also protects again overcorrection due to correlated hypotheses (26).The Neuhaus method was used to correct the misclassification bias on the basis of known sensitivity (98%) and specificity (95%) values of anti-HCV tests (27). We used backward stepwise elimination method to specify the final model.

## 4. Results

The mean age of the sample was 38.71 (median= 38) years for HIV infected persons, and 33.2 (median = 33) for their main partners. One hundred sixty five (98.2%) of couples relation were legal. The prevalence of anti-HCV was 87.5% for HIV infected persons, and 9.5% for their main partners; the mean duration of sexual relationship for couples was 11.9 (median = 8.5) years. Other individual and couple level characteristics of sample are shown in Table 1.

**Table 1.** Characteristics of HIV Infected Persons and their Main Heterosexual Partners, Shiraz (Southern of Iran), 2011

Characteristics	HIV infected persons (Actors)	Partners	Dyads	P value ^a^
**Age, Mean ± SD, y**	38.71 ± 7	33.2 ± 6.28		< 0.0001
**Sex, female/male**	2/166	166/2		< 0.0001
**Age of first drug use, Mean ± SD, y**	17.8 ± 4.54	23.82 ± 6.69		< 0.0001
**Age of first injection of drug, Mean ± SD, y**	25.02 ± 6.25	35.5 ± 9.18		< 0.0001
**Current drug use, No. (%)**	161 (95.8)	10 (6)		< 0.0001
**Injection drug use, No. (%)**	128 (76.2)	6 (3.6)		< 0.0001
**Syringe sharing, No. (%)**	108 (64.3)	3 (1.8)		< 0.0001
**Razor sharing, No. (%)**	109 (64.9)	24 (14.3)		< 0.0001
**Tattooing, No. (%)**	126 (75)	18 (10.7)		< 0.0001
**Homosexual activity, No. (%)**	16 (9.52)	0 (0)		< 0.0001
**Number of sexual contact with main heterosexual partner in last month, Mean **±** SD**			3.7 ±3	
**Duration of sexual relationshipyears, Mean **±** SD**			11.91 ± 8.02	
**Age difference between men and women, y, Mean **±** SD**			6.35 ± 5.06	
**Legally married, No. (%)**			150 (98.2)	
**High education, No. (%)**	24 (14.3)	50 (29.8)		< 0.0001
**Employed fulltime, No. (%)**	118 (70.2)	19 (11.3)		< 0.0001
**HCV status, No. (%)**				
Concordant negative			21 (12.5)	
Concordant positive			16 (9.52)	
Actor positive/partner negative			131 (77.98)	
Actor negative/female positive			0 (0)	
**HIV status, No. (%)**				
Concordant negative			0 (0)	
Concordant positive			77 (46.95)	
Actor positive/ partner negative			91 (53.05)	
Actor negative/ partner positive			0 (0)	

^a^Holm-Sidak adjusted P value

Pairwise correlation was 0.123 (95% CI: 0.09- 0.16), and we considered anti-HCV status between persons within couples as interdependence, and used multilevel approach for the analysis of data.

In univariate multilevel logistic regression analysis, and after adjustment for family-wise error, it was revealed that 11 actor-level (level 1) risk behaviors ( Current drug use, IDU, High risk IDU, Razor sharing, Razor sharing with friends, Razor sharing in prison, Tattooing, the number of tattoo points, Having sex with causal partner, The number of causal partners in past 6 months, The number of sexual contacts with causal partners in past 1 month), 6 actor- level (level 1) cofactors (Age, Sex, CD4 level, HIV positivity, Having job, life time incarceration ), 7 partner-level (level 1) cofactors (CD4 level, History of genital ulcer in previous year, Sexual transmission disease in previous year, HIV positivity, Having job, and High education level), and 2 theory-derived interaction terms (Actor IDU, moderated by actor lifetime incarceration ; and Actor tattooing, moderated by actor HIV positivity) were associated with actor HCV status (Table 2).

**Table 2.** Multilevel Logistic Regression Estimates of Risk Factors/Cofactorsand Theory -Derived Interaction Effects on Actor Anti-HCV Positivity ^a ^, Shiraz (southern of Iran), 2011

	HCV State		OR (95% CI)
	Negative	Positive	
**Risk Behavior (Actor- level, level 1)**			
**Current drug use**			
No	160 (62)	98 (38)	3.5 (2.01- 6.1)
yes	13 (16.7)	65 (83.3)	
**Injection drug use**			
No	164 (81.2)	38 (18.8)	60.3 (27.4- 126.5)
yes	9 (6.7)	125 (93.3)	
**Syringe sharing**			
No	168 (74.7)	57 (25.3)	47.5 (19.9- 113.3)
yes	5 (4.5)	106 (95.5)	
**Razor sharing**			
No	149 (73.4)	54 (26.6)	7.3 (4.4- 12.1)
yes	24 (18)	109 (82)	
**Tattooing**			
No	146 (76)	46 (24)	9.8 (5.5- 16.3)
yes	27 (18.8)	117 (81.2)	
Number of tattooing points	0.37 (0)	2.1 (1)	1.9 (2.3- 1.5)
**Sexual activity with non main heterosexual partner**			
No	153 (66.5)	77 (33.5)	4.9 (2.9- 8.3)
yes	18 (17.3)	86 (82.7)	
**Number of sexual activity with non main heterosexual partner in last month**	0 (0)	0.35 (0)	23.4 (23.01- 23.7)
**Cofactors (Actor- level, level 1)**			
**Age, years**	33.4 ± 6.75	38.66 ± 7.08	1.7 (1.1- 2.4)
**Sex (female, 0; male, 1)**			
Female	152 (90.5)	16 (9.5)	66.5 (33.31- 132.8)
male	21 (12.5)	147 (87.5)	
**CD4 level**	298 ± 187.6	585 ± 279.8	0.996 (0.995- 0.997)
**HIV state**			
Negative	84 (96.6)	3 (3.4)	20.1 (8.8- 48.4)
Positive	86 (35.1)	159 (64.9)	
**Had stable job**			
No	33 (24.1)	104 (75.9)	3.9 (2.4- 6.2)
yes	140 (70.4)	59 (29.6)	
**Lifetime incarceration (at least 7 days)**			
No	163 (86.2)	26 (13.8)	198.3 (21.1- 1863.1)
yes	10 (6.8)	137 (93.2)	
**Cofactors (Partner- level, level 1)**			
**Ever had sexual transmitted disease**			
No	143 (69.4)	63 (30.6)	4.4 (2.7- 7.2)
yes	33 (27.7)	86 (72.3)	
**HIV state**			
Negative	165 (68.5)	7 (31.5)	5.56 (3.3- 10)
Positive	11 (12.6)	76 (87.4)	
**Stable job**			
No	61 (31)	136 (69)	0.17 (0.11- 0.28)
yes	118 (87.4)	17 (12.6)	
**Lifetime incarceration (at least 7 days)**			
No	136 (93.2)	10 (6.8)	46.7 (23.5- 93.1)
yes	43 (23.1)	143 (76.9)	
**couple-level, level 2**			
**No condom use in sexual activity with main heterosexual partner **	42 (25)		1.46 (1.458- 1.462)
**Theory-derived interaction effects**			
**Actor IDU moderated by Actor lifetime incarceration**			0.019 (0.002- 0.18)
**Actor tattooing moderated by Actor HIV positive**			23.42 (23.11- 23.73)

^a^Other independent variables which had not significant association (P > 0.2) with actor HCV status (were not show in this table) are as follows: Actor level: Condom use in sexual activity with non-main heterosexual partner, Current condom use with casual partner, drug use at sexual activity with non-main heterosexual partner time, history of blood transfusion, history of blood transfusion before 1992, number of blood transfusion, age of the first sexual activity, age of the first sexual activity with main heterosexual partner, age of the first drug use, age of the first IDU, total leukocyte count, sexual transmitted disease, education level, homosexual activity Partner level: age, age of the first sexual activity, age of the first drug use, age of the first IDU, CD4 level, total leukocyte count, education level Couple level: razor sharing, drug use at sexual activity with main heterosexual partner time, number of sexual activities with main partner in previous month, Duration of sexual relationship, age difference between them Theory-derived interaction effects: Actor IDU, moderated by Actor HIV positive, sex; Actor tattooing moderated by sex, Actor lifetime incarceration; Sexual activity with non main heterosexual partner moderated by Actor HIV positive, sex; Actor no. of sexual acts with main partner in previous month moderated by Actor HIV positive, Partner HCV positive, Partner HIV positive.

After assessing multi collinearity between those covariates, we included the full model (Table 3). In multivariate analysis the final model showed that actor risk factor of IDU (AOR= 13.03; 95% CI: 3.9- 43.82) and actor cofactors of HIV positivity (AOR = 7.1; 95% CI: 1.37- 36.97), razor sharing (AOR = 4.81; 95% CI: 1.84- 12.55), sex (AOR = 8.83; 95% CI: 3.16- 24.87), and no condom use in sexual activity with main partner (AOR = 6.6; 95% CI: 1.1- 39.6) were associated with actor HCV positivity.

**Table 3.** Multilevel Logistic Regression Estimation of Individual and Couple Level Risk Factors for Actor Anti-HCV Positivity, Final Model, Shiraz (southern of Iran), 2011

Effects ^a^	β	SE	Adjusted Odds Ratio (95% CI)
**Actor IDU**	2.57	0.62	13.03 (3.9-43.82)
**Actor Razor sharing**	1.57	0.49	4.81 (1.84-12.55)
**Actor HIV positive**	1.96	0.84	7.1 (1.37-36.97)
**Actor male**	2.18	0.52	8.83 (3.16-24.78)
**No condom use in sexual activity with main heterosexual partner**	1.89	0.92	6.6 (1.1-39.6)

^a^Random effect (level 2 variance component)

In the next step instead of actor level of IDU, we separately entered 4 actor risk factors (actor current drug use, actor high risk IDU, actor tattooing, and actor lifetime incarceration) in the model that collinearity exists between them. These analyses showed that actor high risk IDU (AOR = 11.1; 95% CI: 2.01- 60.3), and actor lifetime incarceration (AOR = 2.07; 95% CI: 1.16- 3.67) were associated with actor HCV positivity.

## 5. Discussion

In the present study, the possibility of HCV transmission between the partners was shown by determining the association between the individuals and their partners getting infected by HCV. Moreover, the possibility of sexual transmission of HCV was determined by showing the reduction of the risk of HCV transmission between the individuals and their partners in case they used condoms during their sexual relationships. Therefore; in addition to having the risk factors and the cofactors, individuals might get infected by HCV through their partners.

The coinfection of HIV infected individuals and their partners by HCV was 78.4% and 9.5%, respectively in this study. In general the prevalence of HCV-HIV coinfection goes upto 75% in populations that HIV is mainly transmitted by IV drug use and blood transfusion, (28). Since in Iran HIV is transmitted in 70% to 76% cases by intravenous route (29), then high prevalence for this coinfection is expected (30).

Furthermore, different studies conducted on the issue have shown that the rate of intra familial HCV transmission is quite high in the areas with a low prevalence (up to 1%) of the infection (like Iran), and quite low in the regions with a high prevalence of the infection (like Egypt)(31). The possibility of intra familial HCV transmission was also confirmed in the study conducted by de Waure et al. which reviewed 25 studies performed in the areas with a low prevalence of the infection (31). Similar results were also obtained in the study conducted in Georgia, as well (32); however, the studies performed in Iran have revealed quite contradictory results overall (33-35), the possibility of HCV transmission between the individuals and their partners seems to be related to the prevalence of the virus in each region (31), cultural as well as social features of the communities, rate of sharing the instruments in families, and the method used to control both individual, and intra familial risk factors in designing and analyzing the performed studies (6, 36-39).

In general, determining the possibility of virus transmission through sexual route is far more complex in comparison to non-sexual routes because in addition to the above-mentioned factors, the role of intra familial risk factors, such as using shared razor blades or syringes, must be controlled, as well (6, 38). Although a lot of studies have internationally investigated the possibility of HCV transmission among the partners, a limited number of studies have investigated this issue in Iran (33, 35). The study by Mcmahon et al. is one of the most accurate studies on the issue which was conducted in the international level using the same method used in the present study (7). Both Mcmahon’s study and the present one have shown the association between the individuals and their partners being infected by HCV; however, whether this association is due to the intra familial sexual behaviors or risky drug use behaviors is different in the two studies. In addition, regardless of the studies’ methodologies, models, statistical analyses, sampling methods, data collection, and the collected risk factors, the two studies seem to be considerably different regarding the population under study and some of the studied risk factors. In the study by Mcmahon et al. only 20% of the cases were legal spouses and they had averagely been with each other for 7.3 years. In the present study, on the other hand, 150 couples (98.2%) had been legally married and had averagely lived with each other for 11.91 years. Consequently, in that study, the role of intra familial risk factors was limited to a number of particular risk factors, while all the intra familial risk factors were of equal importance in the present study. Furthermore, Ackerman et al. performed a meta-analysis and revealed the importance of length of marriage in HCV transmission (40); nevertheless, using shared razor blades, using condoms, drug use during the intercourse, and the number of intercourses were not investigated in that study. If they had studied these important risk factors, they might have obtained a quite different final model. Overall, considering the fact that most HCV-infected women around the world have no individual risk factors for getting infected by the virus, and their main risk factor is having sexual relationships with infected individuals, this transmission route is of utmost importance (32). In fact, transmission of HCV through the sexual route has been confirmed in a great number of studies (41-45). Moreover, several studies have emphasized the inter partner transmission of HCV in the HIV-infected individuals, which has been shown in a recent meta-analysis conducted by Tohme et al., as well (4).

HIV increases susceptibility and infectiousness of HCV (46). In HIV infected subjects cellular immunologic response in peripheral blood (47) and mucosal cells (48) reduces and therefore the probability of HCV acquisition increases. Beside the probability of spontaneous clearance is reduces too (49).

The present study had some limitations: 1- Drug use and particularly its injection form and having sexual relationships with other partners, particularly for women, are considered as a stigma in Iran; therefore, they might not have been truly reported by the study subjects. 2- The data of the present study were collected in a cross-sectional manner and, as a result, causal inferences regarding HCV transmission could not be obtained. 3- We used ELISA diagnostic screening test in this study for HCV infections, while no confirmatory tests were used. In spite of the high sensitivity of the 3rd generation ELISA tests, this might have led to the misclassification bias which, we hope, has not affected the relationships obtained from the study. Of course, the standard error of the measured parameters was adjusted by using Neuhaus algorithm in the statistical analyses. 4- In this research, no phylogenetic studies were performed on the genetic similarity of the HCV viruses separated from the individuals and their partners. Therefore, HCV transmission between HCV-positive couples could not be definitely determined. On the other hand phylogenetic study does not obviate the role of careful epidemiological analysis (4).

In spite of the above-mentioned limitations, the present study had important strong points, as well. For instance, the risk factors of HCV infection were investigated in both individual and intra familial levels through the APIM model and multilevel analysis. Although this method had been previously used in a study conducted on street addicts, it was used in the HIV-infected population for the first time. Moreover, in addition to taking the intra familial risk factors into account, the present study revealed the role of the intra familial risk factors in relation to sexual issues. This study also investigated the "couples being each other real partners", which was not taken into account in the previous studies. Furthermore, the present study was conducted on a population which plays a key role in HCV transmission through the third wave of HIV infection in Iran. Having a cohort population is considered as another strong point of this study, which helped the researchers to select a control group from the same population employed for selecting the study samples.

To control HCV infection in the infected population and, at the same time, control its transmission to the general population, the following strategies have been proposed: developing a unit regional protocol based on the experiences of different countries for performing frequent serial liver enzyme and HCV tests for the HIV-infected individuals, creating profiles for the spouses or the main partners of the HIV-infected individuals, completing the data collection and risk factors forms when the individuals infected by HIV refer to the centers and upgrade them in defined time intervals, following up the HIV-infected individuals covered by the centers to determine their relationships with their partners and providing them with behavioral care, conducting educational programs to enhance the knowledge as well as the attitude of at risk individuals toward HCV, continually supervising the performance of risk reduction programs in jails as well as other centers, and active long-term follow-up of the released prisoners.

The present study was among the small number of studies conducted to simultaneously investigate individual and dyadic risk factors of HCV infection in the HIV-infected individuals as well as their partners using multilevel analysis. In the present study, the possibility of dyadic HCV transmission was confirmed by showing the internal correlation between the individuals and their partners being infected by HCV. Moreover, the probability of HCV transmission through the sexual route was determined by showing the effect of using condoms in long-term sexual relationships in reducing the risk of getting infected by HCV. Health care providers need to pay special attention to sexual transmission of HCV among HIV-infected individuals, and a periodical screening test seems to be necessary.
